# An interactive analysis of the mouse oviductal miRNA profiles

**DOI:** 10.3389/fcell.2022.1015360

**Published:** 2022-10-19

**Authors:** Angela Taraschi, Costanza Cimini, Alessia Colosimo, Marina Ramal-Sanchez, Luca Valbonetti, Nicola Bernabò, Barbara Barboni

**Affiliations:** ^1^ Faculty of Biosciences and Technology for Food, Agriculture and Environment, University of Teramo, Teramo, Italy; ^2^ Istituto Zooprofilattico Sperimentale Dell’Abruzzo e Del Molise “G. Caporale”, Teramo, Italy; ^3^ Institute of Biochemistry and Cell Biology (CNR-IBBC/EMMA/Infrafrontier/IMPC), National Research Council, Rome, Italy

**Keywords:** miRNA, gene, oviduct, fertilization, embryo development, extracellular vesicles, reproduction, miRnome

## Abstract

MicroRNAs are small non-coding molecules that control several cellular functions and act as negative post-transcriptional regulators of the mRNA. While their implication in several biological functions is already known, an important role as regulators of different physiological and pathological processes in fertilization and embryo development is currently emerging. Indeed, miRNAs have been found in the oviductal fluid packaged within the extracellular vesicles, which might act as natural nanoshuttles by transporting lipids, proteins, RNA molecules and miRNAs from the oviduct to the gametes or embryos. Here, an exhaustive bibliography search was carried out, followed by the construction of a computational model based on the networks theory in an attempt to recreate and elucidate the pathways potentially activated by the oviductal miRNA. The omics data published to date were gathered to create the Oviductal MiRNome, in which the miRNA target genes and their interactions are represented by using stringApp and the Network analyzer from Cytoscape 3.7.2. Then, the hyperlinked nodes were identified to investigate the pathways in which they are involved using the gene ontology enrichment analysis. To study the phenotypical effects after the removal of key genes on the reproductive system and embryo, knockout mouse lines for every protein-coding gene were investigated by using the International Mouse Phenotyping Consortium database. The creation of the Oviductal MiRNome revealed the presence of important genes and their interactions within the network. The functional enrichment analysis revealed that the hyperlinked nodes are involved in fundamental cellular functions, both structural and regulatory/signaling, suggesting their implication in fertilization and early embryo development. This fact was as well evidenced by the effects of the gene deletion in KO mice on the reproductive system and embryo development. The present study highlights the importance of studying the miRNA profiles and their enormous potential as tools to improve the assisted reproductive techniques currently used in human and animal reproduction.

## Introduction

In mammals, the oviduct provides a favourable microenvironment for several events related to fertilization, as the sperm acquisition of fertilizing ability (the capacitation) (Gadella and Boerke, 2016), the sperm-egg recognition and binding (Coy et al., 2012) and the first phases of the embryo development (Li and Winuthayanon, 2017). It exerts its function either by direct or indirect mechanisms: while the oviductal epithelial cells (OECs) directly interact with the spermatozoa storing them in the so-called “functional sperm reservoir” until ovulation (Suarez, 2016), the key molecules contained in the oviductal fluid (OF) contribute to sustain and drive the biochemical machinery of spermatozoa and early embryo development (Avilés et al., 2010; Coy and Yanagimachi, 2015). In this regard, the OF is a complex mixture of molecules that are either passively or actively transported over the epithelial barrier from the circulating blood or the interstitial tissue, or *de novo* secreted by the OECs (Saint-Dizier et al., 2020). It is mainly composed by aminoacids, energy sources , inorganic salts, glycosaminoglycans and numerous proteins (Ballester et al., 2014; Coy and Yanagimachi, 2015; Canha-Gouveia et al., 2019). In particular, the two most abundant proteins identified are the oviduct-specific glycoprotein (OVGP1) and albumin (Mondéjar et al., 2012; Canha-Gouveia et al., 2019), with important roles mainly for sperm capacitation and embryo development. Growth factors are also key proteins of the OF, which exert an activity of cell division control and contribute to the efficient development of early embryos (Pillai et al., 2017). Other components of the OF include low molecular weight hormones such as steroids (progesterone and estradiol) and prostaglandins, which are secreted by the ovarian follicles and the corpus luteus and can reach the OF *via* a local countercurrent transfer (Einer-Jensen and Hunter, 2005). Among these lipid-based molecules stands up progesterone, which is known to play a key role in multiple sperm capacitation events as hyperactivation (Fujinoki et al., 2016), chemotaxis (Teves et al., 2006; Guidobaldi et al., 2008; Oren-Benaroya et al., 2008), induction of acrosome reaction (Therien and Manjunath, 2003; Baldi et al., 2009) and *in vitro* capacitation (Foresta et al., 2009; López-Torres and Chirinos, 2017). Furthermore, steroid hormones and prostaglandins may participate in the transport of gametes and embryos by modulating both muscular contractility and ciliary beat frequency in the oviduct (Ezzati et al., 2014).

During the last years, an additional and more complex component has been identified within the OF. Extracellular vesicles (EVs), also known as oviductosomes (OVS) (Gross et al., 2017; Fereshteh et al., 2018), are released by epithelial cells to the oviductal lumen, being thus classified as exosomes (30–100 nm) or microvesicles (>100 nm) based on their size (Almiñana et al., 2018). They carry bioactive molecules such as lipids, proteins, mRNA and microRNAs (miRNAs) (Asaadi et al., 2021) that can be delivered and fuse with gametes or embryo regulating their functions and interactions and participating in the maternal-embryo communication (Almiñana and Bauersachs, 2019; Bauersachs et al., 2020; Bridi et al., 2020; Harris et al., 2020).

Since the information available on this regard is still poor, here we followed a computational strategy using the networks theory in an attempt to reconstruct the pathways potentially activated by the oviductal miRNA. To this aim, we gathered the omics data published to date to create the Oviductal MiRNome, in which are represented the miRNA target genes and their interactions. The network obtained and the subsequent analysis allowed us to infer important information, useful to improve our knowledge on the potential role of miRNAs in the reproductive events.

## Materials and methods

### Data collection, network construction and analysis

To create the network representing the murine oviductal miRNome (MiRNome and MiRnome_MC), we collected recent data regarding the mirRNome from published peer-reviewed international manuscripts included in Scopus (https://www.scopus.com; accessed on 14/12/2021). To date, only one study systematically analysing the oviductal miRNA profile in the mouse model has been published (Fereshteh and coll, 2018, PMID: 30382141 (Fereshteh et al., 2018)). For each differentially expressed miRNA, we identified the target genes using miRDB (http://mirdb.org/, last accessed on 10/06/2022), an online database for miRNA target prediction and functional annotations (Chen and Wang, 2020). The identified target genes were selected for a target score >90 and submitted to network creation in stringApp for Cytoscape 3.7.2 (Doncheva et al., 2019). The interaction provided by the stringApp were filtered for the *Mus musculus* species, adopting a confidence score of 0.700. The main topological parameters of the network (Table 1) were analysed using Network Analyzer, a plugin of Cytoscape 3.7.2.

**TABLE 1:** Topological parameters of the network. The table shows the definitions of the main topological parameters assessed in this study.

Parameter	Definition
Connected component	Number of networks in which any two vertices are connected to each other by links and which is connected to no additional vertices in the network
Number of nodes (N)	Total number of elements involved within the network
Number of edges	Total number of interactions among the nodes within the network
Clustering coefficient	It is calculated as CI = 2nI/(kI − 1), where nI is the number of links connecting the kI neighbors of node I to each other. It is a measure of how the nodes tend to form clusters
Network diameter	The longest of all the calculated shortest paths in a network
Shortest paths	Length of the shortest path between two nodes n and m is (n, m). The shortest path length distribution gives the number of node pairs (n, m) with (n, m) = k for k = 1, 2,
Characteristic path length	Expected distance between two connected nodes
Averaged number of neighbors	Mean number of connections of each node
Node degree (k)	Number of interactions of each node
Node degree distribution (P(k))	Probability that a selected node has k links
Μ	Exponent of node degree equation
R^2^	Coefficient of determination of node degree vs. number of nodes, on logarithmized data

In keeping with Bernabò et al. (Ordinelli et al., 2018; Bernabò et al., 2019), we identified the hubs within Murine Oviductal MiRNome as the nodes with a degree at least one standard deviation above the network mean. In details, the hubs were identified based on the following formula:

ND>μ+σ

where:

ND =: node degree
μ =: averaged node degree
σ =: standard deviation of node degree.

### Enrichment analysis

We carried out an enrichment Gene ontology (GO) characterizes the relationship between genes by specifically annotating and categorizing the molecular function of a gene product, the associated biological process and the cellular component, referring to the place in the cell where a gene product performs a function (Ashburner et al., 2000). We used The Gene Ontology Consortium’s online tool (http://www.geneontology.org/, last accessed on 10/06/2021) for the enrichment analysis of our hubs list, selecting as species *Mus musculus*.

### Identification of phenotypical effects of the deletion of genes relative to murine oviductal MiRNome hubs in KO mice in the reproductive system and embryos

To study the phenotypical effects after the removal of key genes (*i.e.,* genes codifying for the murine oviductal MiRNome hubs) on the reproductive system and embryo, we used the International Mouse Phenotyping Consortium (IMPC) (https://www.mousephenotype.org/), a portal that provides a freely available comprehensive catalogue of mammalian genes function, by producing a knockout mouse line for every protein-coding gene.

## Results

### Murine oviductal MiRNome creation, analysis and visualization

**FIGURE 1:**
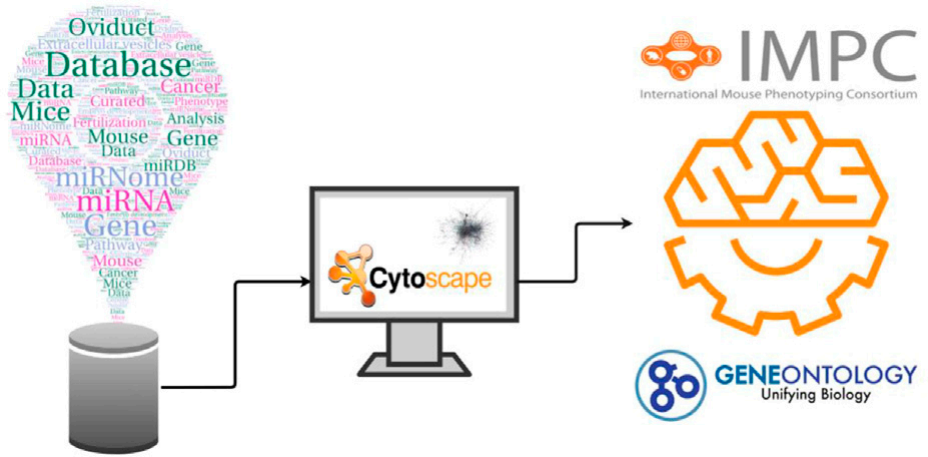
Experimental design. The figure illustrates the steps followed during the development of the work. 1) we collected the oviductal miRNA from the literature and we obtained from the miRDB their experimentally verified targets. 2)The identified target genes were submitted to network creation in stringApp for Cytoscape 3.7.2.3) We performed an enrichment Gene Ontology and used the International Mouse Phenotyping Consortium to investigate the phenotypic impact on the reproductive system and embryo after the removal of important genes.

**FIGURE 2:**
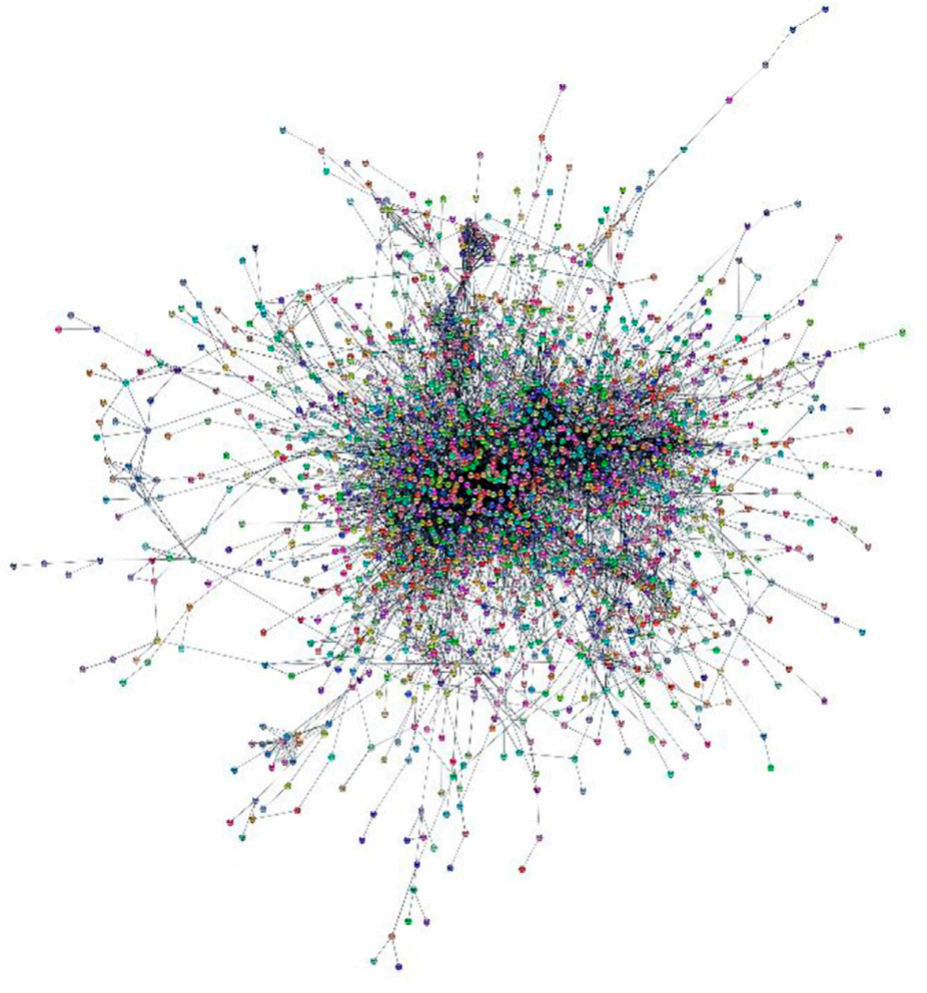
MiRNome_MC network. The figure shows the MiRNome Main Component (MiRNome_MC). The network was created with Cytoscape 3.7.2.

The experimental design, including all the steps for the creation and analysis of the network, is illustrated in Figure 1. We collected the experimentally validated targets for the gathered oviductal miRNAs from miRDB. A total of 2,689 miRNA-target were collected from mouse and were used to build the Murine Oviductal MiRNome. Specifically, the network obtained is undirected and composed by 850 connected components, 2,665 nodes and 6,599 links. Since the largest connected component has 1746 nodes, representing 65.5% of Murine Oviductal MiRNome, all the further analysis were carried out on this network, called MiRNome Main Component, MiRNome_MC (Figure 2).

The values of its main topological parameters are listed in Table 2 and in Supplementary Material 1.

**TABLE 2:** Main topological parameters computed on MiRNome and MiRNome_MC networks.

Parameters	MiRNome	MiRNome_MC
Number of nodes	2,665	1746
Number of edges	6,599	6,529
Connected components	850	1
Clustering coefficient	0.203	0.310
Diameter	15	15
Shortest paths	3,046,966 (42%)	3,046,770 (100%)
Charact. path length	4.669	4.670
Avg. number of neighbours	4.952	7.479

The node degree distribution follows a power law, characterized by a negative exponent (–1.702) and it is with the node degree (R2 = 0.1989). Thus, the MiRNome_MC does not possess an evident hierarchical pattern and it can be considered as a Barabasi—Albert network (Table 3).

**TABLE 3:** Node degree distribution in MiRNome_MC. The node degree distribution represents the probability that a selected nodes has k links (γ = exponent of node degree equation; R^2^ = coefficient of determination of node degree vs. number of nodes, on logarithmized data).

Node degree distribution	Value
Γ	-1.702
R	0.817
R^2^	0.904

### Identification of hubs and gene ontology enrichment analysis

We found a total of 179 hubs in MiRNome_MC (the complete list of hubs and the topological parameters are available in the supplementary material 1), representing almost the 10% of the nodes (179 out of 1746). Then, we recorded the biological processes in which the hubs are engaged using gene ontology (GO) annotation, such as metabolic processes, gene expression, cell signaling, cell cycle and death. Most importantly, several miRNA targets were associated with GO categories “reproduction (GO:0000003)”, “developmental process involved in reproduction (GO:0003006)”, “embryo development (GO:0009790)” and “embryonic morphogenesis (GO:0048598)” (supplementary material 2).

And 13 genes were found respectively on reproductive system and embryo phenotype of mice KO models studies among the 179 hubs

By evaluating the effects derived from the depletion of genes among the 179 hubs of the Murine Oviductal MiRNome_MC, we found that 11 genes have been studied on mice KO models due to their relationship with the reproductive system (Table 4 and supplementary material 3), while 13 genes were the focus of works related to the embryo phenotype (Table 5 and supplementary material 3). Among these miRNAs target genes, three of them, namely *Afdn, Itpr1* and *Med1*, were found to be related to both analyzed functions in knock-out mice (i.e., reproductive system and embryo phenotype), while only *Rac1* showed a much greater number of links with respect to the other genes (93 *versus* 30-17).

**TABLE 4:** Genes studied in mice KO models in terms of their relationship with the reproductive system. The table shows the number of links for each gene, the genes and the protein for which codifies, the phenotype in mice with the corresponding MGI (mouse genome informatic database accession number), the role in human diseases and their corresponding references. PCOS: Polycystic ovary syndrome; ALL: Acute lymphocytic leukemia; MLL: mixed lineage leukemia; AML: acute myeloid leukemia; MPPH: Megalencephaly-polymicrogyria-polydactyly-*hydrocephalus*.

N of links	Gene	Protein	Phenotype in mice	MGI accession number	Role in human diseases	References
**93**	*Rac1*	Rac family small GTPase	Abnormal testis morphology; small testis	97845	Germline mutations causative of MRD48; Somatic mutations involved in cardiovascular, cerebrovascular, renal and cardiac diseases; Proto-oncogene (glioblastoma, melanoma, leukemia, brain, lung, testicular and breast cancers)	Nagase and Fujita, 2013; Carrizzo et al., 2014; Feng et al., 2021
**38**	*Itpr1*	Inositol 1,4,5-trisphosphate receptor 1	Small epididymis	96623	Germline mutations causative of Gillespie syndrome, SCA15 and SCA29; Proto-oncogene (multiple myeloma, breast, lung and renal carcinomas)	Whaley et al., 2011; Messai et al., 2014; Gerber et al., 2016; Alfugham et al., 2018
**36**	*Ppp1cc*	Protein phosphatase 1 catalytic subunit gamma	Male infertility	104872	Proto-oncogene (malignant fibrous histiocytoma, osteogenic and soft tissue tumors)	Yamada et al., 1994; Sogawa et al., 1996; Chakrabarti et al., 2007; MacLeod and Varmuza, 2012
**32**	*Ube2n*	Ubiquitin conjugating enzyme E2 N	Abnormal seminal vesicle morphology	1934835	PCOS; transsexuality; Proto-oncogene (melanoma, colorectal, cervical, ovarian carcinomas and others)	Pulvino et al., 2012; Cheng et al., 2014; Wu et al., 2014; Dikshit et al., 2018; Gemoll et al., 2019; Song et al., 2020; Dong et al., 2021
**27**	*Med1 (Mbd4)*	Mediator complex subunit 1	Enlarged uterus	1100846	Hepatic autophagy; Tumor suppressor gene (colorectal, gastric, endometrial, pancreatic cancers)	Howard et al., 2009; Lucci-Cordisco and Neri, 2009; Leonard and Zhang, 2019
**26**	*Ppp2r1b*	Phosphatase 2 scaffold subunit Abeta	Male infertility	1,920,949	Azoospermia; Tumor suppressor gene (lung, colon, endometrial carcinomas and others)	Wang et al., 1998; Sablina et al., 2007; Tzur et al., 2009; Remmerie and Janssens, 2019
**24**	*Plcb1*	Gene phospholipase C beta 1	Male infertility; female infertility; abnormal ovary morphology	97613	Germline mutations causative of DEE12 and MMPEI; Proto-oncogene (cholangiocarcinoma, colorectal, hepatocellular, ovarian cancers)	Lu et al., 2019; Lin et al., 2020; Liang et al., 2021
**20**	*Afdn (Mllt4)*	Adherens junction formation factor	Abnormal uterus morphology	1,314,653	Tumor suppressor gene (ALL); Proto-oncogene (endometrial, gastric, colon cancers and others)	Takai and Nakanishi, 2003; Sun et al., 2014; Yamamoto et al., 2015; Lai et al., 2020
**18**	*Skp2*	S-phase kinase associated protein 2	Male infertility; Female infertility	1,351,663	Proto-oncogene (osteosarcoma, lymphomas, colorectal, breast and prostate cancers)	Hershko, 2008; Katoh et al., 2013; Asmamaw et al., 2020; Shi et al., 2021; Wu et al., 2021
**18**	*Sacm1l*	SAC1 like phosphatidylinositide phosphatase	Enlarged epididymis; abnormal testis morphology; small testis; abnormal epididymis morphology	1,933,169	Associated to COVID-19 severity*;* MLL–SACM1L rearrangement in absence of leukemia	Mori et al., 2010; Wu et al., 2021
**18**	*Ccnd2*	Cyclin D2	Small testis	88314	Germline mutations causative of MPPH3; Tumor suppressor gene (lymphomas; AML) and proto-oncogene (ovarian, testicular, breast, colorectal cancers and others)	Sicinski et al., 1996; Mirzaa et al., 2012; Chen et al., 2018; Jardim et al., 2021

**TABLE 5:** Genes studied in mice KO models in terms of their relationship with the embryo phenotype. The table shows the number of links for each gene, the genes and the protein for which codifies, the phenotype in mice with the corresponding MGI (mouse genome informatic database accession number), the role in human diseases and their corresponding references. ALL: Acute lymphocytic leukemia; AML: acute myeloid leukemia.

N of links	Gene	Protein	Phenotype in mice	MGI accession number	Role in human diseases	References
**38**	*Itpr1*	Inositol 1,4,5-trisphosphate receptor 1	Embryonic growth retardation; abnormal embryo size	96623	Germline mutations causative of Gillespie syndrome, SCA15 and SCA29; Proto-oncogene (multiple myeloma, breast, lung and renal cancers)	Whaley et al., 2011; Jayadev and Bird, 2013; Messai et al., 2014; Gerber et al., 2016; Alfugham et al., 2018
**27**	*Med1 (Mbd4)*	Mediator complex subunit 1	Abnormal placenta size; abnormal embryo size	1100846	Hepatic autophagy; Tumor suppressor gene (colorectal, gastric, endometrial, pancreatic cancers)	Bellacosa, 2001; Howard et al., 2009; Leonard and Zhang, 2019
**24**	*Ap2b1*	Adaptor-related protein complex 2, beta-1 subunit	Abnormal embryo size	1,919,020	Tumor suppressor gene (triple-negative breast cancer); Proto-oncogene (prostate and chemioresistant ovarian cancers)	Cheng et al., 2010; Rangel et al., 2017; Kaikkonen et al., 2020; Fang et al., 2021
24	*Ubxn7*	UBX domain protein 7	Abnormal embryo size; embryonic growth retardation	2,146,388	Proto-oncogene (lungs squamous cell carcinoma)	Wang et al. (2013)
21	*Cacna1c*	Calcium voltage-gated channel subunit alpha1 C	Abnormal placenta morphology	103013	Germline mutations causative of Brugada syndrome, Romano-Ward syndrome and Timothy type 1 syndrome); Polymorphisms associated to neuropsychiatric disorders; Proto-oncogene (leukemia, breast, brain tumors); Tumor suppressor gene (ovarian and endometrial cancers)	Splawski et al., 2004; Fukuyama et al., 2014; Wang et al., 2015; Gourraud et al., 2017; Moon et al., 2018; Gardner et al., 2019; Qiao et al., 2019; Chang and Dong, 2021
**21**	*Plod2*	Procollagen-lysine,2-oxoglutarate 5-dioxygenase 2	Abnormal embryo size	1,347,007	Germline mutations causative of Bruck syndrome; Proto-oncogene (breast,.colorectal. lung, bladder, cervical, ovarianrenal, and bone cancers)	Hu et al., 2019; Du et al., 2020; Wan et al., 2020; Wei et al., 2021
**20**	*Afdn (MIIt4)*	Adherens junction formation factor	Abnormal neural tube morphology; abnormal neural tube closure	1,314,653	Tumor suppressor gene (ALL); Proto-oncogene (endometrial, gastric, colon cancers and others)	Takai and Nakanishi, 2003; Sun et al., 2014; Yamamoto et al., 2015; Lai et al., 2020
**20**	*Gna13*	G protein subunit alpha 13	Abnormal visceral yolk sac morphology; abnormal embryo size	95768	Proto-oncogene (B-cell lymphoma; ovarian, prostate, colorectal and gastric cancers and others)	Guney et al. (2022)
**20**	*Ncoa2*	Nuclear receptor coactivator 2	Abnormal umbilical cord morphology; abnormal placenta vasculature	1,276,533	Translocations in various cancers (AML, ALL; mesenchymal chondrosarcoma); Proto-oncogene (prostate cancer)	Taylor et al., 2010; Leiner and Le Loarer, 2020
**19**	*Arhgef12*	Rho guanine nucleotide exchange factor 12	Abnormal embryo size; embryonic growth retardation	1,916,882	Glaucoma; Tumor suppressor gene (AML, lymphomas, pancreatic cancer)	Springelkamp et al., 2015; Yang et al., 2020; Panagopoulos et al., 2021
**19**	*Bmi1*	Polycomb ring finger	Abnormal embryo size	88174	Proto-oncogene (breast, gastric, ovarian, lung, pancreatic cancers and others)	Liu et al., 2017; Janaki Ramaiah and Vaishnave, 2018; Zhao et al., 2018; Wang et al., 2019; Chen et al., 2021
**18**	*Dnmt3a*	DNA methyltransferase 3 alpha	Abnormal embryo size	1,261,827	Germline mutations causative of Tatton-Brown-Rahman syndrome and Sporadic pheochromocytoma/secreting paraganglioma; Tumor suppressor gene (AML, leukemia and other hematologic cancers); Proto-oncogene (testicular tumor)	Chen and Chan, 2014; Brunetti et al., 2017; Bullinger et al., 2017
**17**	*Acvr2a*	Activin A receptor type 2A	Abnormal embryo size	102806	Susceptibility to preeclampsia	Glotov et al., 2019; Pinyol et al., 2021
					Tumor suppressor gene (hepatocellular carcinoma)	

## Discussion

The potential role of miRNAs in the regulation of genes during fertilization and early embryo development has recently attracted the attention of several researchers (Gross et al., 2017; Reza et al., 2019; Salilew-Wondim et al., 2020). MicroRNAs are small (22 nucleotides) non-coding molecules involved in the control of cellular functions and that act generally as negative post-transcriptional regulators of mRNA (Lau et al., 2001). They have been shown to participate in numerous biological processes, including gametogenesis and embryo development (Salilew-Wondim et al., 2020), and are present in several tissues of the reproductive system such us testis, epididymis, spermatozoa and seminal plasma (Salas-Huetos et al., 2020), evincing their strong involvement in the reproductive field. Moreover, microRNAs are emerging as regulators of different physiological and pathological processes in fertilization and embryo development (Hossain et al., 2012; Gross et al., 2017; Tesfaye et al., 2018; Salilew-Wondim et al., 2020). Interestingly, they have been discovered also in the oviductal fluid (Almiñana et al., 2018; Fereshteh et al., 2018; Gonella-Diaza et al., 2021; Mazzarella et al., 2021), packaged within the extracellular vesicles (EVs, also called oviductosomes, OVS) (Gross et al., 2017; Fereshteh et al., 2018) that might act as natural nanoshuttles, bringing key components (lipids, proteins, RNA molecules and miRNAs) from the oviduct to the gametes or the embryos (Almiñana et al., 2018). As a result, the OVS may encapsulate miRNAs, preventing degradation and increasing their stability in OF. The oviductal EVs may then release their contents into the embryo *via* mechanisms such as membrane fusion or endocytosis. However, it is still unknown whether these vesicles can use more than one route or if vesicular uptake is cell type specific (Pavani et al., 2017). Little is known regarding their biogenesis but that are transcribed as primary miRNAs (pri-miRNAs) (Lee et al., 2002) and processed to precursor miRNAs (pre-miRNA) by the nuclear RNase III, DROSHA (Ha and Kim, 2014; Leitão and Enguita, 2022). Pre-miRNAs are then transferred to the cytoplasm, where an RNase III endonuclease DICER cleaves the pre-miRNA to the mature form of the miRNA (Ha and Kim, 2014). The mature miRNA generated by DICER is chained with Argonaute (AGO) protein to form an effector complex called RNA-induced silencing complex (RISC) able to exert its regulatory action (Ha and Kim, 2014; Leitão and Enguita, 2022). However, little is still known about the biological functions of miRNAs expressed in the EVs.

Here, we adopted a computational biology approach gathering the available data to reconstruct the pathway that may be activated by the oviductal miRNA in a mouse model. Mouse (*Mus musculus*) is the most commonly used animal model in biomedical research, including reproductive biology (Jamsai and O’Bryan, 2011; Ramal-Sanchez et al., 2021). Furthermore, the mouse model allows the assessment of IVF rates and early stages of embryo development, as well as the ascertainment of the resulting offspring in terms of health status and potential epigenetic modifications. Most importantly, because of their high genetic similarity to humans and ease of genetic manipulation, mice models are largely used to study the vast majority of human diseases (Rosenthal and Brown, 2007; Perlman, 2016). Nowadays, many types of mouse models, such as knockout/knockin, transgenic and chemical-mutagenized mutant mouse models have been made available not only for biomedical research but also to reveal disease-associated genes, including causes of male infertility (Jamsai and O’Bryan, 2011). Specifically, we realized the network representing the murine oviductal miRNome (MiRNome and MiRnome_MC), with the available published omic data. Once the network model was obtained, we assessed its topology to infer important biological information. As evident for the parameters listed in Table 3, the miRNome is a scale-free network that follows the Barabasi-Albert (BA) model. It means that the node degree distribution follows a power-law with a negative exponent and is not correlated with the clustering coefficient (Albert and Barabasi, 2002; Ravasz and Barabási, 2003). Thus, it is possible to identify a low number of highly connected nodes (the “hubs”) coexisting with a higher number of scarcely connected nodes (Barabási and Oltvai, 2004). These networks are characterized by a high robustness against the random damages and by an efficient information transfer. In particular, the hubs exert a significant control over the whole network, assuring that the information is spread within the network in a very robust, fast and efficient way and that the network is able to quickly response to internal and external stimuli (Albert and Barabasi, 2002; Ravasz and Barabási, 2003; Barabási and Oltvai, 2004).

In this regard, first we carried out the identification of the hyperlinked nodes and then investigated the pathway in which they are involved using the gene ontology enrichment analysis. In general, the functional enrichment analysis revealed that these genes codify for proteins that are involved in fundamental cellular functions, both structural (membrane, cell junction and cytoskeleton organization, collagen synthesis) and regulatory/signaling (cell cycle regulation, signal transduction, transcriptional activation, endocytosis, calcium channel activity, ubiquitination, dephosphorylation, methylation, chromatin repression), by referring to 4 and 18 genes, respectively. All these roles suggest their implication in fertilization and early embryo development, as evidenced by the effects of the gene deletion in KO mice on the reproductive system and embryo development (Tables 4 and 5). Our findings are consistent with previous research, since Fereshteh and coll. (2018) have demonstrated that murine EVs can deliver miRNAs to the sperm cells, such as miR-34c-5p, which is located in the sperm centromere and promotes the first zygote cleavage (Fereshteh et al., 2018). At the same time, murine EVs contain other miRNAs that may target several embryonic development-related genes (Fereshteh et al., 2018). *In vitro*, EVs supplementation altered the bovine transcriptome, implying that oviductal EVs miRNA cargo may have a potential role in controlling embryonic development (Bauersachs et al., 2020). Furthermore, it has been recently showed that the expressed miRNA in bovine EVs modulate different pathways, including PI3K/AKT, mTOR and MAPK, which are related to transcription, translation, proliferation, growth, control of the cytoskeletal organization and metabolism, and that may influence the early embryo development within the oviduct (Mazzarella et al., 2021). PI3K/AKT/mTOR signaling pathways also regulates angiogenesis (Karar and Maity, 2011), a crucial process for the proper functioning of the female reproductive system and for pregnancy establishment (Rizov et al., 2017). Additionally, several reports have suggested that the deletion of miRNA processing genes may have a serious effect on reproductive functions or embryo development, mostly due to altered miRNA levels (Kaczmarek et al., 2020). Notably, the loss of *Dicer1* and *Ago2* in mice are embryonically lethal (Bernstein et al., 2003; Yang et al., 2005; Alisch et al., 2007). Among others, the deficiency of *Dicer* in the mouse reproductive tract hampered uterine development, resulting in pregnancy loss after wild-type embryo transfer (Gonzalez and Behringer, 2009). Thus, microRNAs are involved in several processes affecting the function of the reproductive system, as well as the conception, the implantation process, and the embryonic development.

Noteworthy, all events in the oviduct are orchestrated by the neuroendocrine axis through the dynamic changes induced by steroid hormones. In a recent study, Almiñana and coll. (2018) showed interesting differences in the miRNAs content and the protein composition of oviductal extracellular vesicles isolated from cows at various stages of the estrous cycle, suggesting that ovarian steroid hormones may regulate the EVs production and secretion (Almiñana et al., 2018). These changes, directed by the neuroendocrine axis, are reflected on the early embryonic genome reprogramming. In fact, the delicate process known as the maternal-zygotic transition (MZT) occurs in the oviduct (Baroux et al., 2008). In mammalian pre-implantation embryos, the maternal gene products regulate the initial events of embryogenesis, while the zygotic genome remains transcriptionally silent (Baroux et al., 2008; Hamm and Harrison, 2018). Developmental control is then passed from the mother to the zygote (Hamm and Harrison, 2018). In addition, a recent comprehensive characterization of the bovine OF proteome revealed a spatiotemporal regulation of the OF according to the anatomical region of the oviduct, the proximity to the ovulating ovary and the stage of the cycle (Mahé et al., 2022). Future approaches should investigate the potential involvement of miRNA in the fine-tune regulation of the oviductal microenvironment.

Importantly, miRNAs play a significant role in reproduction and may represent excellent research candidates with the potential to improve the understanding of the complex landscape in which fertilization occurs, as well as the underlying molecular mechanisms that prevent implantation and embryo progression. This knowledge may be useful in developing new *in vitro* fertilization (IVF) systems that mimic the physiological condition as closely as possible. Indeed, the current IVF systems lack the interaction of gametes with several components naturally present in the reproductive tract during fertilization and the early stages of development, that may be responsible for the impaired *in vitro* development and viability, but also for some epigenetic changes in *in vitro*-produced embryos, resulting in imprinting disorders such as Beckwith-Wiedemann Syndrome (BWS) and Angelman syndrome (AS) (Harris et al., 2020). Therefore, detailed knowledge on oviductal transcriptome and secretome are needed to develop better embryo culture medium and conditions in order to reduce the adverse periconceptional environment in *in vitro* derived embryos.

Interestingly, 7 out of the 21 protein-coding genes studied here (*RAC1, ITPR1, PLCB1, CACNA1C, PLOD2, CCND2, DNMT3A*) are causative of inherited monogenic diseases when germinally mutated in humans, and all of them (with the possible exclusion of *SACM1L)*, result to be deregulated in several types of human cancers when somatically mutated. As such, they can be classified as proto-oncogenes (*RAC1, ITPR1, PPP1CC, UBE2N, PLCB1, UBXN7, PLOD2, GNA13, NCOA2, BML1, SKP2),* tumor suppressor genes (*MED1, PPP2R1B, ARHGEF12, ACVR2A)* or both *(AP2B1, CCANA1C, AFDN, CCND2, DNMT3A)* (Tables 4 and 5). This last observation highlights the importance of oviductal miRNAs in acting themselves as tumor suppressor genes or oncogenes, depending on the cancer type and cellular context (Barbato et al., 2017; Ghafouri-Fard et al., 2020).

The most prominent role as proto-oncogenes and the fact that at least 9 of these oviductal miRNA-target genes (*PPP1CC, PPP2R1B, CCND2, ACVR2A*, *MED1, NCOA2, PLCB1, ARHGEF12, GNA13)* are involved in proliferation regulatory pathways (i.e. cell cycle regulation, transcriptional activation, signal transduction) may explain the observed effect of their deletion in KO mice on embryo development, suggesting as the embryo could be the mainly sensitive target of their action.

Regarding the reproductive system, three of these genes (*UBE2N, PPP2R1B* and *ACVR2A*) have been shown to be involved in reproductive disfunctions in humans (polycystic ovary syndrome and transsexuality, azoospermia, and susceptibility to preeclampsia, respectively (Fitzpatrick et al., 2009; Dong et al., 2021; Du et al., 2021). So far, a total of 15 genes out of 21 (*UBE2N, PPP2R1B, CACNA1C, AP2B1, NCOA2, RAC1, MED1, PLOD2, GNA13, AFDN, CCND2, BMI1, PLCB1, DNMT3A* and *SKP2)* have been associated to tumors of the female or male reproductive tracts (cervical carcinoma, ovarian, endometrial, prostate, and testicular cancers (Tables 4 and 5), in addition to other types of cancers or diseases. All the human orthologs genes have been better described in Supplementary material 4, including their functional role and implications in human diseases, both inherited and acquired.

These results support the idea that understanding the role of oviductal miRNAs in human cancer pathogenesis could be fundamental for their application as biomarkers and as potential therapeutic options for malignancies treatments. More specifically, a recent *in vitro* study has shown the involvement of 20 specific miRNAs in promoting the development and progression of ovarian carcinoma, the most aggressive and hard-to-detect gynecological cancer worldwide (Loginov et al., 2022). More recently, the protective role exerted by two miRNAs, miR-145 and miR-93-5p, in suppressing ovarian cancer cell proliferation and migration through a negative transcriptional regulation of the proto-oncogene *CCND2* have been described in two different studies (Hua et al., 2019; Chen et al., 2022)**.**

In conclusion, the adoption of a biological network-based approach allowed us to infer new and interesting processes involved in fertilization and in the early embryo development, demonstrating the utility of computational modelling strategies in the reproductive field. Our data clearly revealed that the targets of the miRNAs are involved in processes critical for fertilization and early embryo development. Interestingly, data from the KO mice model seem to support the biological relevance of our findings; nevertheless, more functional experiments are required to further our understanding of the role of oviductal miRNA in fertilization or early embryo development. However, we highlight here the importance of studying the miRNA profiles and their enormous potential as tools to improve the assisted reproduction techniques (ARTs) currently used in human and animal reproduction and as biomarkers or potential therapeutic options for malignancies treatments in humans.

## Data Availability

The original contributions presented in the study are included in the article/Supplementary material, further inquiries can be directed to the corresponding author.
