# Supplementary material for: An interactive analysis of the mouse oviductal miRNA profiles
Source: Front Cell Dev Biol. 2022 Oct 19;10:1015360. doi: 10.3389/fcell.2022.1015360 (PMC9627480; doi:10.3389/fcell.2022.1015360)
Supplement: Supplementary file 3 [file DataSheet1.docx]

**Supplementary Material 4**. List of human genes from Tables 4 and 5 with their implications in human diseases. Genes are presented in alphabetical order: *ACVR2A; AFDN; AP2B1; ARHGEF12; BMI1; CACNA1C; CCND2; DNMT3A; GNA13; ITPR1; MED1; NCOA2; PLCB1; PLOD2; PPP1CC; PPP2R1B; RAC1; SACM1L; SKP2; UBE2N; UBXN7.*

- ***ACVR2A* (activin A receptor type 2A) (OMIM *102581)**

This gene encodes a receptor that mediates the functions of activins, which are members of the transforming growth factor-beta (TGF-beta) superfamily involved in diverse biological processes. In culture, this TGF-β-related activin receptor functionally acts as a tumor suppressor gene in hepatocellular carcinoma (HCC) (Pinyol *et al.*, 2021). Concordantly, *in vivo* studies have shown significantly higher rates of mutations in *ACVR2A* (10%) in patients with HCC associated with non-alcoholic steatohepatitis (NASH), compared to viral/alcohol-HCC (3%) (Pinyol *et al.*, 2021).

Some polymorphic variants in the *ACVR2A* gene have been associated with susceptibility to preeclampsia (PE), a pregnancy-related disease which can result in maternal and fetal morbidity and mortality (Glotov *et al.*, 2019). Nevertheless, further studies are needed to reveal the role of this gene in the pathogenesis of PE during pregnancy*.*

- ***AFDN (AFADIN)* (Adherens junction formation factor) (OMIM *159559)**

The *AFDN* gene encodes a multi-domain scaffold protein involved in signaling and organization of cell junctions during embryogenesis. Afadin is commonly found in both adherens and tight junctions, playing structural and signal-modulating roles. As such, it is involved in several cellular processes implicated in cancer progression, including signal transduction, migration, invasion and apoptosis. Due to its pleiotropic functions, *AFDN* is considered both a tumor suppressor gene (fusion partner of the *ALL-1* gene, involved in acute myeloid leukemias) and a proto-oncogene in gastric (Lai *et al.*, 2020), colon (Sun *et al.*, 2014) and endometrial cancers (Yamamoto *et al.*, 2015). Furthermore, afadin connects the intercellular adhesion molecule nectin to the actin cytoskeleton. This intercellular adhesion system is involved in the formation of synapses in neurons and the organization of heterotypic junctions between Sertoli cells and spermatids in the testis (Takai and Nakanishi, 2003).

- ***AP2B1* (adaptor-related protein complex 2, beta-1 subunit) (OMIM *601025)**

The protein encoded by this gene is the beta subunit of the assembly protein complex 2, which serves to link clathrin to receptors in coated vesicles to mediates clathrin-dependent endocytosis. Mice that carry the homozygous mutation (p.M448K) exhibit cardiac phenotypes of DORV, AVSD, and arch anomalies, while the extra-cardiac phenotypes of homozygous mutant mice include micrognathia, cleft palate, and thymus hypoplasia, reminiscent of neural crest anomalies. In human, overexpression of *AP2B1* in triple-negative breast cancer suggested that this downstream target of Zinc Fingers and Homeoboxes 2 (ZHX2) contribute to the oncogenic role of *ZHX2* in an accumulative fashion (Fang *et al.*, 2021). Another study showed that *AP2B1,* together with *ADNP, TOMM70A* and *ZNF326*, showed tumor suppressor activity in triple-negative breast cancer xenograft studies (Rangel *et al.*, 2017). In addition, AP2B1 results to be an interacting protein of anoctamin 7 (ANO7) in prostate cancer cells (Kaikkonen *et al.*, 2020). A previous study that used a next-generation sequencing analysis in ovarian cancer cell lines and tissues with different chemotherapy response phenotypes revealed a subnetwork with three genes *BMP7*, *NR2F2* and *AP2B1* that were consistently overexpressed in the chemoresistant ovarian tissue or cells (Cheng *et al.*, 2010), thus confirming the role as tumor suppressor gene of *AP2B1.*

- ***ARHGEF12* (Rho guanine nucleotide exchange factor 12) (OMIM *604763)**

The protein encoded by this gene belongs to a family of guanine nucleotide exchange factors (GEFs) that activate small GTPases of the Rho family and catalyze the exchange of GDP for GTP. Rho GTPases play a fundamental role in several cellular processes by forming a complex with G proteins and stimulating Rho-dependent signals. In humans, *ARHGEF12* plays an important role in the RhoA/RhoA kinase pathway, which has been implicated in intraocular pressure (IOP) regulation, being an important risk factor for most types of glaucoma, with both mendelian and complex inheritance. A previous study has shown a specific role of *ARHGEF12* in increasing the risk of primary open-angle glaucoma, the most common adult-onset form of glaucoma with complex inheritance (Springelkamp *et al.*, 2015).

The ARHGEF12 protein has also been observed to form a myeloid/lymphoid fusion partner in acute myeloid leukemia (Yang et al 2020), while a KMT2A-ARHGEF12 fusion protein has been described in a child with a high-grade B-cell lymphoma (Panagopoulos *et al.*, 2021). A recent study has shown the role of *Arhgef12* as proto-oncogene in pancreatic ductal adenocarcinoma. The transcriptional repression of *Arhgef12*, *RhoA*, and *Sdc4* mediated by miR-802 has been associated with the suppression of pancreatic cancer initiation (Ge *et al.*, 2022).

- ***BMI1* (polycomb ring finger) (OMIM *164831)**

The *BMI1* gene encodes a ring finger protein that is major component of the polycomb group complex 1 (PRC1). This complex functions as an epigenetic chromatin repressor of several genes involved in embryonic development and in different fundamental cellular processes including cell senescence, apoptosis and somatic stem cell renewal and differentiation. *Bmi1*-knockout resulted in complete infertility in female mice by increasing oxidative stress and DNA damage, inhibiting granulosa cell proliferation, and inducing granulosa cell apoptosis (Wang *et al.*, 2019).

Since the polycomb group complex exerts a fundamental role in DNA damage repair, the *BMI1* gene is considered a proto-oncogene. Its altered expression is causative of numerous cancers (including breast, gastric, ovarian, lung, pancreatic) (Liu *et al.*, 2017; Janaki Ramaiah and Vaishnave, 2018; Zhao *et al.*, 2018; Chen *et al.*, 2021), and is associated with resistance to certain chemotherapies.

- ***CACNA1C* (calcium voltage-gated channel subunit alpha1 C) (OMIM *114205)**

This gene encodes an alpha-1 subunit of a voltage-dependent calcium channel that mediate the influx of calcium ions into the cell upon membrane polarization. The alpha-1 subunit consists of 24 transmembrane segments and forms the pore through which the ions pass into the cell.

Germline mutations of this gene have been associated to different genetic cardiac disorders: Brugada Syndrome 3 (BRGDA3), Romano-Ward syndrome and Timothy Syndrome.

Brugada syndrome 3 (OMIM #611875; ORPHA:130),) is a rare inherited arrhythmia syndrome, characterized by specific electrocardiogram pattern (so-called type 1 ECG) and leading to an increased risk of sudden cardiac death in patients with structurally normal hearts (Gourraud et al., 2017). The syndrome typically manifests during adulthood, with a mean age of sudden death of 41 +/- 15 years, but also occurs in infants and children.

Romano-Ward syndrome (RWS) (OMIM #192500) is a form of familial long QT (LQT) syndrome, inherited as autosomal dominant, characterized by syncopal episodes and electrocardiographic abnormalities (QT prolongation, T-wave abnormalities and torsade de pointes (TdP) ventricular tachycardia). RWS may result from mutations in genes either encoding subunits of cardiac ion channels (*CACNA1C*, *KCNH2, SCN5A, KCNE1, KCNE2* and *SCN4B*)*,* or proteins interacting with cardiac ion channels (*ANK2, CAV3, AKAP9 o SNTA1*). The terms of LQT1-LQT6 and LQT9-LQT12 describe patients with genetic variants of RWS.

Timothy Syndrome (TS) (OMIM #601005; ORPHA:595098), also known as long QT syndrome with syndactyly (LQT8), is characterized by multiorgan dysfunction, including lethal arrhythmias, webbing of fingers and toes, congenital heart disease, immune deficiency, intermittent hypoglycemia, cognitive abnormalities, and autism (Splawski *et al.*, 2004).This cardiac arrhythmia may result in recurrent syncope, seizure, or sudden death. In the 5-generation family reported by (Gardner *et al.*, 2019), the transmission pattern of LQT8 was consistent with autosomal dominant inheritance with incomplete penetrance.

In addition, single-nucleotide polymorphisms (SNPs) in *CACNA1C* have been consistently associated with an increased risk for a broad spectrum of neuropsychiatric disorders, including schizophrenia, bipolar disorder and major depression (Moon *et al.*, 2018).

Different meta-analysis results showed that *CACNA1C* was highly expressed in leukemia, brain, bladder and breast cancer and other tumors, suggesting its regulatory roles as proto-oncogene in cancer progression (Wang *et al.*, 2015). The same gene is also considered a tumor suppressor gene because somatic mutations in *CACNA1C* have been significantly associated with longer overall survival time in patients with ovarian cancer (Chang and Dong, 2021) and with endometrial cancer, one of the most common gynecologic malignancies (Qiao *et al.*, 2019). In endometrial cancer tissue, bioinformatics analysis showed that *CACNA1C* is involved in regulating collagen fibril organization, cell-matrix adhesion, cellular response to amino acid stimulus, cell adhesion, and negative regulation of cell proliferation (Qiao *et al.*, 2019).

- ***CCND2* (Cyclin D2) (OMIM *123833)**

The *CCND2* gene encodes Cyclin D2, a member of the conserved cyclin family, that functions as regulator of CDK4 or CDK6 kinases. Cyclin D2 is required for cell cycle G1/S transition and has been shown to be involved in the phosphorylation of tumor suppressor protein Rb. Knockout mice of this gene have demonstrated the essential roles of *ccnd2* in ovarian granulosa and germ cell proliferation. Germline *de novo* mutations in *CCND2* are causative of a malformation syndrome called megalencephaly-polymicrogyria-polydactyly-hydrocephalus syndrome 3 (MPPH3)**.**

The MPPH3 disorder (ORPHA:83473) is due to gain-of-function *CCND2* mutations causing the expansion of neuronal progenitor cells and the clinical phenotype observed in affected individuals: macrocephaly, megalencephaly, ventriculomegaly, polymicrogyria, polydactyly and severely delayed psychomotor development (Mirzaa *et al.*, 2012).

In accordance with the critical role in cell cycle regulation, *CCND2* exhibits both tumor suppressive and pro-metastatic functions. In fact, in hematopoietic malignancies, translocations involving the *CCND2* gene have been found in lymphomas while mutations in the same gene have been reported in AML. As proto-oncogene, *CCND2* overexpression has been reported in several solid tumors (ovarian, testicular, breast, colorectal cancers and others) (Sicinski *et al.*, 1996; Jones and Vasey, 2003; Chen *et al.*, 2018; Gu *et al.*, 2018; Jardim *et al.*, 2021).

- ***DNMT3A* (DNA methyltransferase 3 alpha) (OMIM *** **602769)**

This gene encodes a DNA methyltransferase that is thought to function in *de novo* CpG methylation, an epigenetic modification that is important for embryonic development, imprinting, X-chromosome inactivation, aging and cancer. The protein localizes to the cytoplasm and nucleus and its expression is developmentally regulated. Studies in mice have demonstrated that DNA methylation is required for mammalian development. Germline mutations in *DNMT3A* are causative of Tatton-Brown-Rahman syndrome (ORPHA:404443), a rare multiple congenital anomalies syndrome characterized by greater height, mild to moderate intellectual disability and distinctive facial appearance (Tovy *et al.*, 2022). Germline mutations are also causative of Sporadic pheochromocytoma/secreting paraganglioma (ORPHA:276621), a rare, isolated, non-familial pheochromocytoma/paraganglioma tumor arising from neuroendocrine chromaffin cells of the adrenal medulla (pheochromocytoma) or from extra-adrenal chromaffin tissue (paraganglioma) (Remacha *et al.*, 2018).

The role of *DNMT3A* as a proto-oncogene has been confirmed by the finding of several somatic mutations in a large variety of immature and mature hematologic neoplasms (Ferris *et al.*, 2022). These mutations seem to confer poor prognosis to acute myeloid leukemia (AML) patients, showing a prevalence of *DNMT3A* mutations in 12–35% of the cases (Brunetti *et al.*, 2017)*.*  However, recent studies have identified several inactivating mutations of *DNMT3A* in myeloid malignancies, as well as a loss of DNMT3A activity at advanced tumor stages, suggesting a more complex role of DNMT3A in tumorigenesis. This gene can act as a proto-oncogene in some circumstances, but also seems to behave as a tumor suppressor in other cases (Chen and Chan, 2014).

Different studies have shown the dual roles of DNMT3A in cancer suppression or growth promotion, much depending on which miRNA is controlling the *DNMT3A* mRNA expression. For example, miR-29 family members target DNMT3A in lung cancer (Fabbri *et al.*, 2007), and AML (Garzon *et al.*, 2009), while miR-199a-3p targets the DNMT3A2 isoform in testicular cancer (Chen *et al.*, 2014).

- ***GNA13*** (**G protein subunit alpha 13) (OMIM *604406)**

The *GNA13* gene encodes a Guanine nucleotide binding protein (G protein) subunit that is predicted to enable D5 dopamine receptor binding activity and to have a GTPase activity. This protein is involved in several processes, including Rho protein signal transduction, activation of phospholipase D activity, and multicellular organism aging. It is also involved in blood vessel morphogenesis, negative regulation of vascular associated smooth muscle cell migration, and negative regulation of vascular associated smooth muscle cell proliferation. In mouse, disruption of the *Gna13* gene impaires the ability of endothelial cells to develop into an organized vascular system, resulting in intrauterine death and demonstrating the role of GNA13 in developmental angiogenesis.

In humans, *GNA13* is upregulated in many solid tumors and impacts survival and metastases in patients affected with B-cell lymphomas (Guney *et al.*, 2022) ovarian (Yagi *et al.*, 2016) and prostate cancers (Rasheed *et al.*, 2013), colorectal and gastric cancers (Zhang *et al.*, 2016a, 2018), hepatocellular carcinoma (Xu *et al.*, 2016) and head and neck squamous cell carcinoma (Rasheed *et al.*, 2018), among others.

- ***ITPR1* (Inositol 1,4,5-trisphosphate receptor 1) (OMIM *147265)**

The *ITPR1* gene encodes the inositol 1,4,5-trisphosphate receptor that, upon stimulation, modulates calcium ions release from endoplasmic reticulum. These receptors are located in neuronal and nonneuronal tissues and are involved in several processes, including epithelial fluid transport, apoptotic signaling pathway, calcium ion transport, endoplasmic reticulum calcium ion homeostasis and voluntary musculoskeletal movement. Germline mutations in the *ITPR1* gene cause at least three different neurological conditions: Gillespie syndrome, spinocerebellar ataxia type 15/16 and spinocerebellar ataxia type 29, according to the type and location of the mutations.

Gillespie syndrome (GS) (ORPHA:1065) is mostly inherited in an autosomal recessive pattern and is characterized by bilateral aniridia, congenital hypotonia, non-progressive ataxia, cerebellar atrophy, delayed motor developmental and mild to moderate intellectual disability. At least 13 *ITPR1* gene mutations have been identified in individuals with Gillespie syndrome, including homozygous partial deletions, compound heterozygous truncating mutations or heterozygous mutations located within the transmembrane domain (Gerber *et al.*, 2016). However, the specific correlation between these sequence variations and the clinical phenotype of patients affected with GS remains unclear. Spinocerebellar ataxia type 15/16 (SCA15) (ORPHA:98769) is a very slowly progressive form of pure cerebellar ataxia with onset in adulthood and is inherited as an autosomal dominant disorder. Only seven families have been identified worldwide in which different heterozygous complete or partial deletions of *ITPR1* have been reported (Whaley *et al.*, 2011). Unlike SCA29 and GS, it is not typically associated with delayed motor development or intellectual disability.

Spinocerebellar ataxia type 29 (SCA29) (ORPHA:208513) is a congenital cerebellar ataxia type I due to germline mutations in the *ITPR1* gene. SCA29 is inherited autosomal dominantly, occasionally autosomal recessively, and is characterized by very slowly progressive or non-progressive ataxia, dysarthria, oculomotor abnormalities and intellectual disability (Jayadev and Bird, 2013).

Somatic mutations in the *ITPR1* gene have been implicated in tumor growth promotion, classifying it as a proto-oncogene. An association of ITPR autoimmunity with neurological conditions and several types of cancer (breast, lung carcinoma and multiple myeloma) has been described in 8 patients (Alfugham *et al.*, 2018). One study reported that an ITPR1 upregulation in a renal cell carcinoma related to von Hippel-Lindau syndrome was associated to resistance to conventional anticancer treatment (Messai *et al.*, 2014).

- ***MED1* (Mediator complex subunit 1) (OMIM *604311)**

The *MED1* gene (also called *MBD4*- methyl-CpG binding domain 4, DNA glycosylase) encodes for a multiprotein coactivator that is required by DNA-binding transcription factors for polymerase II-transcribed genes*.* MED1 is also a component of other multiprotein complexes such as thyroid hormone receptor-(TR-) associated proteins. As a transcriptional enhancer, it is also involved in p53-dependent apoptosis, is essential for adipogenesis and plays an essential role in regulating hepatic autophagy and lipid oxidation.

As a DNA glycosylase in base excision repair (BER) system, MBD4 interacts with the mismatch repair (MMR) protein MLH1, suggesting that these two proteins act in the same apoptotic pathway. *MED1* is considered a tumor suppressor gene, since frameshift mutations in this gene have been reported in human colorectal, gastric, endometrial, pancreatic (Bellacosa, 2001) and breast cancers (Leonard and Zhang, 2019). Its inactivation may contribute to tumorigenesis, acting as a modifier of mismatch repair-defective colorectal tumors, likely by increasing the genomic instability phenotype (Lucci-Cordisco and Neri, 2009).

In absence of known genetic mutations, *MED1* epigenetic promoter methylation and consequent decreased gene expression have been associated with the initiation of sporadic colorectal and epithelial ovarian cancers rather than tumor progression (Howard *et al.*, 2009).

- ***NCOA2* (nuclear receptor coactivator 2) (OMIM *601993)**

The *NCOA2* gene, also known as *GRIP1****,*** encodes for a transcriptional coactivator for nuclear hormone receptors, including steroid, thyroid, retinoid and vitamin D receptors. The protein acts as an intermediary factor for the ligand-dependent activity of these nuclear receptors, which regulate their target genes upon binding of cognate response elements. *NCOA2* has been found to be involved in chromosomal translocations that result in fusions with other genes in various human cancers, including the vestigial-like 2 (*VGLL2*) gene in rhabdomyosarcoma (Leiner and Le Loarer, 2020), the lysine acetyltransferase 6A (*KAT6A*) gene in acute myeloid leukemia, the ETS variant 6 (*ETV6*) gene in acute lymphoblastic leukemia and the hes related family bHLH transcription factor with YRPW motif 1 (*HEY1*) gene in mesenchymal chondrosarcoma. *NCOA2* somatic mutations have been reported in human melanoma and lung cancer, and a gain of expression of *NCOA2* was identified in 218 human prostate cancer tumors in approximately 11% of samples (Taylor *et al.*, 2010). The high frequency of *NCOA2* overexpression in primary tumors and its known role as an androgen receptor (AR) coactivator suggest that these two genes might collaborate in early prostate cancer progression by enhancing AR transcriptional output and providing a mechanism for potential role of *NCOA2* as a prostate cancer oncogene.

- ***PLCB1* (phospholipase C beta 1) (OMIM *607120)**

The *PLCB1* gene encodes a member of the phospholipase family, the phospholipase C-beta enzyme, that catalyzes the formation of inositol 1,4,5-trisphosphate and diacylglycerol from phosphatidylinositol 4,5-bisphosphate. This enzymatic reaction is a key step in the intracellular transduction of many extracellular signals. The mammalian PLCB1 isoform is expressed in select areas of the brain, including cerebral cortex, hippocampus, amygdala, lateral septum, and olfactory bulb. For this reason, germline mutations in *PLCB1* are causative of two severe neurologic disorders in humans: Developmental and epileptic encephalopathy-12 (DEE12) and Malignant migrating focal seizures of infancy (MMPEI).

DEE12 (OMIM 613722) is an autosomal recessive neurologic disorder characterized by onset of refractory seizures in the first year of life. Affected babies have normal or mildly delayed development before the onset of seizures, but afterwards they show severe developmental regression and stagnation with little or no progress (absent speech, hypotonia, poor motor skills, peripheral spasticity, and impaired visual fixation).

MMPEI (ORPHA:293181) is a rare epileptic and developmental encephalopathy characterized by focal seizure onset during the first months of life, marked drug resistance, and severe, long-term cognitive disability. The genetic etiology is variable with different causative genes (including *KCNT1*, *SCN2A*, *PLCB1* among others) and consequent different mode of inheritance (autosomal dominant, autosomal recessive, or X-linked).

Mutation of the *plcb1* gene in the mouse sperm reduces the acrosome reaction rate, fertilization rate, and embryo development rate, holding central roles in reproductive physiology.

In humans PLCB1 is involved in phospholipid hydrolysis and is frequently upregulated in human cancers, thus functioning as an oncogenic driver in cholangiocarcinoma, colorectal, hepatocellular and ovarian cancers (Lu *et al.*, 2019; Fan *et al.*, 2020; Lin *et al.*, 2020; Liang *et al.*, 2021).

- ***PLOD2* (Procollagen-lysine, 2-oxoglutarate 5-dioxygenase 2) (OMIM *601865)**

The protein codified by *PLOD2* is a membrane-bound enzyme which catalyzes the hydroxylation of lysyl residues in collagen-like peptides. The resultant hydroxylysyl groups are attachment sites for carbohydrates in collagen, making PLOD2 a critical protein for the stability of intermolecular crosslinks in the major component of extracellular matrix. Altered hydroxylation and collagen cross-link supports the progression of many collagen-related diseases, such as fibrosis and cancer.

Germline mutations in the coding region of this gene are associated with Bruck syndrome (ORPHA:2771), a rare autosomal recessive disease characterized by osteogenesis imperfecta and congenital joint contractures.

The overexpression of PLOD2 that is involved in the degradation of procollagen lysine has been associated with various tumor entities, including breast (Hu *et al.*, 2019), colorectal (Du *et al.*, 2020), ovarian (Wei *et al.*, 2021), endometrial (Wan *et al.*, 2020), lung, bladder, cervical, renal and bone cancers (Du *et al.*, 2017), demonstrating the proto-oncogene role of PLODs in promoting cancer progression and metastasis.

- ***PPP1CC* (protein phosphatase 1 catalytic subunit gamma) (OMIM *176914)**

The *PPP1CC* gene encodes for the gamma isoform of a protein belonging to the PP1 subfamily of protein phosphatase. PP1 is a ubiquitous serine/threonine phosphatase that regulates many cellular processes, including cell division. The three closely related isoforms (alpha, beta/delta and gamma) have distinct localization patterns. In addition, the *PPP1CC* gene encodes two alternatively spliced variants, PP1 gamma1 (PPP1CC1) and PP1 gamma2 (PPP1CC2). While PPP1CC1 is ubiquitous in somatic cells, PPP1CC2 is expressed exclusively in testicular germ cells and sperm. Homozygous deletion of the *Ppp1cc* gene, which encodes both isoforms, results in impaired spermiogenesis in knockout male mice (-/-) (MacLeod and Varmuza, 2012). It has been suggested that besides motility, the PPP1CC2 isoenzyme might play a specific function in the development of specialized flagellar structures of mammalian spermatozoa (Chakrabarti *et al.*, 2007). In humans, *PPP1CC* acts as a proto-oncogene being involved in the accelerated growth of tumor cells in malignant fibrous histiocytoma (Yamada *et al.*, 1994), in malignant osteogenic tumors (chondrosarcoma, osteosarcoma, and Ewing's sarcoma) and in malignant soft tissue tumors (liposarcoma and malignant fibrous histiocytoma)(Sogawa *et al.*, 1996).

Among the oviductal miRNA target genes also implicated in human reproductive disfunctions we have found six genes: *UBE2N, PPP2R1B, ACVR2A, CACNA1C, AP2B1,* and *NCOA2.* These genes and their role will be discussed further in detail below.

#### **PPP2R1B (protein phosphatase 2 scaffold subunit Abeta) (OMIM *603113)**

*PPP2R1B,* also known as *PP2AA-BETA***,** encodes the beta isoform of the constant regulatory subunit (PP2AA) of protein phosphatase 2 (PP2A), a heterotrimer composed of a dimeric core enzyme that consists of a scaffold (A) and catalytic (C) subunit and a third variable regulatory subunit (B). PP2A is a serine threonine phosphatase implicated in the negative control of cell proliferation and involved in other fundamental cellular processes, such as signal transduction, DNA repair, and apoptosis. The beta subunit PP2AA-B is necessary for the interaction of the catalytic PP2AC and variable PP2AB subunits and is critical for phosphatase activity in cell proliferation. It has been shown that suppression of *PPP2R1B* expression allowed immortalized human cell lines to achieve a tumorigenic state, concluding that this gene is a tumor suppressor that transforms immortalized cells by regulating the small GTPase RALA function (Sablina *et al.*, 2007). In fact, somatic alterations in this gene have been found in several human malignancies, including breast, lung, skin, lung, colon cancers (Wang *et al.*, 1998) as well as endometrial carcinomas (Remmerie and Janssens, 2019), suggesting that PP2AA-B may suppress tumor development through its role in cell cycle regulation and cellular growth control.

While homozygous deletion of *Ppp2r1b* in mouse impairs meiotic recombination and causes meiotic arrest in spermatocytes, heterozygous missense mutations in the human *PPP2R1B* gene have been first identified in two non-obstructive azoospermic patients belonging to a four- generation family, exhibiting dominant transmission of infertility (Du *et al.*, 2021). The same authors found different heterozygous mutations in other 120 non-related infertile patients with meiotic arrest.

- ***RAC1* (Rac family small GTPase 1) (OMIM *602048)**

The *RAC1* gene (also called *p21RAS*) encodes for a GTPase belonging to the RAS superfamily of small GTP-binding proteins that regulate numerous cellular functions essential for normal development. In particular, RAC1 is an important modulator of the cytoskeleton exerting a critical function in phagocytosis, autophagy, mesenchymal-like migration, neuronal polarization, axonal growth, adhesion and differentiation of multiple cell types (Reijnders *et al.*, 2017). *De novo* germinal heterozygous missense mutations in the *RAC1* gene have been described in seven unrelated boys affected with MRD48 (OMIM #617751), an autosomal intellectual developmental disorder (DD) with varying degrees of developmental delay, brain malformations and additional phenotypes (Reijnders *et al.*, 2017). This rare multiple congenital anomalies/dysmorphic syndrome is also called “Microcephaly-corpus callosum and cerebellar vermis hypoplasia-facial dysmorphism-intellectual disability syndrome” (ORPHA: 500159), and is characterized by global developmental delay and from moderate to severe intellectual disability and other clinical manifestations. Somatic activating mutations in the *RAC1* gene have been implicated in different cardio- and cerebrum-vascular pathologies (Carrizzo *et al.*, 2014), and in renal and cardiac diseases (Nagase and Fujita, 2013). In addition, the modulator function of the RAC1 protein in the autophagy pathway may explain its role as proto-oncogene in promoting glioblastoma development (Feng *et al.*, 2021).

- ***SACM1L* (SAC1 like phosphatidylinositide phosphatase) (OMIM *606569)**

The *SACM1L* gene encodes SAC1, an integral membrane protein of the endoplasmic reticulum and the Golgi apparatus. It functions as a phosphoinositide phosphatase by hydrolyzing phosphatidylinositol 3-phosphate, phosphatidylinositol 4-phosphate and phosphatidylinositol 3,5-bisphosphate. Knockout mice of this gene show preimplantation lethality. Other studies have shown that mutations in the *SACM1L* gene alter SAC1 enzymatic activity and the organization of mammalian Golgi membranes and mitotic spindles apparatus (Liu *et al.*, 2008). A recent study has shown that 10 single nucleotide polymorphisms (SNPs) in the *SACM1L* gene negatively moderated the sexual-side effects of the antidepressant bupropion in the treatment of major depressive disorders (Clark *et al.*, 2012). Since the gene is located within a 250-kb tumor suppressor region on chromosome 3p21.3, a putative role as proto-oncogene has been hypothesized for *SACM1L.* So far, only a case of a 3-years old boy carrying a novel rearrangement between mixed lineage leukemia (MLL) gene and *SACM1L* in the absence of leukemia has been described (Mori *et al.*, 2010). A very recent study has included *SACM1L* among eight causal genes for COVID-19 severity (Wu *et al.*, 2021a).

- ***SKP2* (S-phase kinase associated protein 2) (OMIM *601436)**

The *SKP2* gene encodes a member of the F-box protein family which constitutes one of the four subunits of ubiquitin protein ligase complex called SCFs (SKP1-cullin-F-box). This protein complex functions in phosphorylation-dependent ubiquitination and is an essential element of the cyclin A-CDK2 S-phase kinase. The protein Skp2 is a component of the multisubunit cullin-RING ligase (CRL1) which is expressed in many tissues and participates in multiple cellular functions, including cell proliferation, metabolism, and tumorigenesis by contributing to the ubiquitination and subsequent degradation of several specific tumor suppressors.

To this regard, *SKP2* is established as a proto-oncogene causally involved in the pathogenesis of a plethora of cancers (lymphomas, osteosarcoma, colorectal, breast, prostate) since *SKP2* overexpression enhances cell growth, accelerates cell cycle progression, promotes migration and invasion, and inhibits cell apoptosis (Hershko, 2008; Chan *et al.*, 2010; Katoh *et al.*, 2013; Zhang *et al.*, 2016b; Asmamaw *et al.*, 2020; Shi *et al.*, 2021). The role of *SKP2* in drug resistance and poor prognosis through cell cycle and mitosis regulation, EMT (epithelial-mesenchymal transition) property, enhanced DNA damage response and repair, has been also recently elucidated (Wu *et al.*, 2021b).

- ***UBE2N* (ubiquitin conjugating enzyme E2 N) (OMIM *603679)**

The *UBE2N* gene codes for an E2 ubiquitin-conjugating enzyme that catalyzes lys63-linked polyubiquitin chains for several important proteins. Being involved in ubiquitin modification this protein results fundamental in the cellular mechanism for targeting abnormal or short-lived proteins for degradation. Studies in mouse suggest that this protein plays a role in DNA postreplication repair. In human, *UBE2N* was identified, together to other 4 genes, as the most similarly expressed gene between Polycystic ovary syndrome PCOS and transsexuals (Dong *et al.*, 2021). While PCOS is a common cause of female infertility in which female patients show signs of hyperandrogenism due to high levels of androgens, transsexuals are women showing male signs due to the assumption of androgen drugs for a long time. This study has shown that all the five identified genes are involved in the ubiquitination of androgen receptor and eventually may cause sinus follicular growth arrest.

As proto-oncogene, UBE2N plays significant roles in several tumors including neuroblastoma, B-cell lymphoma, breast, and colorectal cancers (Pulvino *et al.*, 2012; Cheng *et al.*, 2014; Wu *et al.*, 2014; Gemoll *et al.*, 2019). Other studies have shown that *UBE2N* is overexpressed in melanoma cells, playing a significant role in melanoma growth and tumor progression. Its knockdown markedly inhibited cell proliferation and subcutaneous tumor growth of melanoma by suppressing MEK/FRA1/SOX10 signaling pathway (Dikshit *et al.*, 2018). As well, *UBE2N* was found to have an oncogenic role also in cervical carcinoma, since the knockdown of this gene markedly suppressed tumoral cells growth (Song *et al.*, 2020). In that study, *UBE2N* overexpression in cervical carcinoma was inhibited by miR-590-3p, suggesting that targeting the miR-590-3p/UBE2N axis could be a potential strategy for the treatment of cervical carcinoma.

- ***UBXN7* (UBX domain protein 7) (OMIM *616379)**

This gene encodes for UBXN7 (also called UBXD7*)*, a cofactor protein belonging to ubiquitin ligase complexes that participate in the degradation of misfolded or damaged proteins in the p97-mediated ubiquitin proteasome system (UPS). A recent study has shown that knocking down the mRNA expression of four genes located at 3q26-29, including *UBXN7*, led to the inhibition of cell proliferation in amplified lung squamous cell carcinomas (SCC), suggesting a proto-oncogene role for all these genes (Wang *et al.*, 2013).

**References**

Alfugham N, Gadoth A, Lennon VA, Komorowski L, Scharf M, Hinson S, McKeon A, Pittock SJ. ITPR1 autoimmunity: Frequency, neurologic phenotype, and cancer association. *Neurol Neuroimmunol NeuroInflammation* 2018;**5**:418. American Academy of Neurology.

Asmamaw MD, Liu Y, Zheng YC, Shi XJ, Liu HM. Skp2 in the ubiquitin-proteasome system: A comprehensive review. *Med Res Rev* [Internet] 2020;**40**:1920–1949. Med Res Rev.

Bellacosa A. Role of MED1 (MBD4) Gene in DNA repair and human cancer. *J Cell Physiol* [Internet] 2001;**187**:137–144. J Cell Physiol.

Brunetti L, Gundry MC, Goodell MA. DNMT3A in Leukemia. *Cold Spring Harb Perspect Med* 2017;**7**:. Cold Spring Harb Perspect Med.

Carrizzo A, Forte M, Lembo M, Formisano L, Puca A, Vecchione C. Rac-1 as a new therapeutic target in cerebro- and cardio-vascular diseases. *Curr Drug Targets* 2014;**15**:1231–1246. Curr Drug Targets.

Chakrabarti R, Cheng L, Puri P, Soler D, Vijayaraghavan S. Protein phosphatase PP1γ2 in sperm morphogenesis and epididymal initiation of sperm motility. *Asian J Androl* 2007;**9**:445–452.

Chan CH, Lee SW, Wang J, Lin HK. Regulation of Skp2 expression and activity and its role in cancer progression. *ScientificWorldJournal* [Internet] 2010;**10**:1001–1015. ScientificWorldJournal.

Chang X, Dong Y. CACNA1C is a prognostic predictor for patients with ovarian cancer. *J Ovarian Res* 2021;**14**:. J Ovarian Res.

Chen BF, Chan WY. The de novo DNA methyltransferase DNMT3A in development and cancer. *Epigenetics* 2014;**9**:669–677. Epigenetics.

Chen BF, Gu S, Suen YK, Li L, Chan WY. microRNA-199a-3p, DNMT3A, and aberrant DNA methylation in testicular cancer. *Epigenetics* 2014;**9**:119–128. Epigenetics.

Chen C, Xia E, Bhandari A, Wang Y, Shen Y, Sindan N, Lin Y, Wang X, Yang F, Wang O. LncRNA CCND2-AS1 is up-regulated and regulates proliferation, migration, and invasion in breast cancer. *Int J Clin Exp Pathol* [Internet] 2018;**11**:1453. e-Century Publishing Corporation.

Chen MH, Fu LS, Zhang F, Yang Y, Wu XZ. LncAY controls BMI1 expression and activates BMI1/Wnt/β-catenin signaling axis in hepatocellular carcinoma. *Life Sci* [Internet] 2021;**280**:. Life Sci.

Cheng J, Fan YH, Xu X, Zhang H, Dou J, Tang Y, Zhong X, Rojas Y, Yu Y, Zhao Y, *et al.* A small-molecule inhibitor of UBE2N induces neuroblastoma cell death via activation of p53 and JNK pathways. *Cell Death Dis* 2014;**5**:. Cell Death Dis.

Cheng L, Lu W, Kulkarni B, Pejovic T, Yan X, Chiang JH, Hood L, Odunsi K, Lin B. Analysis of chemotherapy response programs in ovarian cancers by the next generation sequencing technologies. *Gynecol Oncol* 2010;**117**:159. NIH Public Access.

Clark SL, Adkins DE, Aberg K, Hettema JM, McClay JL, Souza RP, Oord EJCG Van Den. Pharmacogenomic study of side-effects for antidepressant treatment options in STAR*D. *Psychol Med* 2012;**42**:1151–1162. Psychol Med.

Dikshit A, Zhang JY, Dikshit A, Zhang JY. UBE2N plays a pivotal role in maintaining melanoma malignancy. *Oncotarget* 2018;**9**:37347–37348. Impact Journals.

Dong R, Gao S, Shan MJ. Identification of the similarly expressed genes in patients with polycystic ovary syndrome and transsexuals. *Medicine (Baltimore)* 2021;**100**:e26990. Wolters Kluwer Health.

Du H, Pang M, Hou X, Yuan S, Sun L. PLOD2 in cancer research. *Biomed Pharmacother* [Internet] 2017;**90**:670–676. Biomed Pharmacother.

Du M, Yuan L, Zhang Z, Zhang C, Zhu M, Zhang Z, Li R, Zhao X, Liang H, Li Y, *et al.* PPP2R1B is modulated by ubiquitination and is essential for spermatogenesis. *FASEB J* 2021;**35**:. FASEB J.

Du W, Liu N, Zhang Y, Liu X, Yang Y, Chen W, He Y. PLOD2 promotes aerobic glycolysis and cell progression in colorectal cancer by upregulating HK2. *Biochem Cell Biol* [Internet] 2020;**98**:386–395. Biochem Cell Biol.

Fabbri M, Garzon R, Cimmino A, Liu Z, Zanesi N, Callegari E, Liu S, Alder H, Costinean S, Fernandez-Cymering C, *et al.* MicroRNA-29 family reverts aberrant methylation in lung cancer by targeting DNA methyltransferases 3A and 3B. *Proc Natl Acad Sci U S A* 2007;**104**:15805–15810. Proc Natl Acad Sci U S A.

Fan Y, Wang L, Han XC, Ma HY, Zhang N, Zhe L. LncRNA MIF-AS1 aggravates the progression of ovarian cancer by sponging miRNA-31-5p. *Eur Rev Med Pharmacol Sci* [Internet] 2020;**24**:2248–2255. Eur Rev Med Pharmacol Sci.

Fang W, Liao C, Shi R, Simon JM, Ptacek TS, Zurlo G, Ye Y, Han L, Fan C, Ortiz CL, *et al.* ZHX2 prom 1 otes hif1α oncogenic signaling in triple-negative breast cancer. *Elife* 2021;**10**:. eLife Sciences Publications Ltd.

Feng X, Zhang H, Meng L, Song H, Zhou Q, Qu C, Zhao P, Li Q, Zou C, Liu X, *et al.* Hypoxia-induced acetylation of PAK1 enhances autophagy and promotes brain tumorigenesis via phosphorylating ATG5. *Autophagy* 2021;**17**:723–742. Autophagy.

Ferris MA, Smith AM, Heath SE, Duncavage EJ, Oberley M, Freyer D, Wynn R, Douzgou S, Maris JM, Reilly AF, *et al.* DNMT3A overgrowth syndrome is associated with the development of hematopoietic malignancies in children and young adults. *Blood* 2022;**139**:461–464. Elsevier B.V.

Gardner RJMK, Crozier IG, Binfield AL, Love DR, Lehnert K, Gibson K, Lintott CJ, Snell RG, Jacobsen JC, Jones PP, *et al.* Penetrance and expressivity of the R858H CACNA1C variant in a five-generation pedigree segregating an arrhythmogenic channelopathy. *Mol Genet genomic Med* 2019;**7**:. Mol Genet Genomic Med.

Garzon R, Liu S, Fabbri M, Liu Z, Heaphy CEA, Callegari E, Schwind S, Pang J, Yu J, Muthusamy N, *et al.* MicroRNA-29b induces global DNA hypomethylation and tumor suppressor gene reexpression in acute myeloid leukemia by targeting directly DNMT3A and 3B and indirectly DNMT1. *Blood* 2009;**113**:6411–6418. Blood.

Ge W, Goga A, He Y, Silva PN, Hirt CK, Herrmanns K, Guccini I, Godbersen S, Schwank G, Stoffel M. miR-802 Suppresses Acinar-to-Ductal Reprogramming During Early Pancreatitis and Pancreatic Carcinogenesis. *Gastroenterology* 2022;**162**:269–284. Gastroenterology.

Gemoll T, Miroll E, Klein O, Lischka A, Eravci M, Thorns C, Habermann JK. Spatial UBE2N protein expression indicates genomic instability in colorectal cancers. *BMC Cancer* 2019;**19**:710. NLM (Medline).

Gerber S, Alzayady KJ, Burglen L, Brémond-Gignac D, Marchesin V, Roche O, Rio M, Funalot B, Calmon R, Durr A, *et al.* Recessive and Dominant De Novo ITPR1 Mutations Cause Gillespie Syndrome. *Am J Hum Genet* 2016;**98**:971. Elsevier.

Glotov AS, Kazakov S V., Vashukova ES, Pakin VS, Danilova MM, Nasykhova YA, Masharsky AE, Mozgovaya E V., Eremeeva DR, Zainullina MS, *et al.* Targeted sequencing analysis of ACVR2A gene identifies novel risk variants associated with preeclampsia. *J Matern Neonatal Med* 2019;**32**:2790–2796. Taylor & Francis.

Gourraud JB, Barc J, Thollet A, Marec H Le, Probst V. Brugada syndrome: Diagnosis, risk stratification and management. *Arch Cardiovasc Dis* 2017;**110**:188–195. Arch Cardiovasc Dis.

Gu W, Sun Y, Zheng X, Ma J, Hu XY, Gao T, Hu MJ. Identification of Gastric Cancer-Related Circular RNA through Microarray Analysis and Bioinformatics Analysis. *Biomed Res Int* [Internet] 2018;**2018**:. Biomed Res Int.

Guney E, Lucas C-HG, Qi Z, Yu J, Zhang R, Ohgami RS, Rubenstein JL, Boué DR, Schafernak KT, Wertheim GB, *et al.* A genetically distinct pediatric subtype of primary CNS large B-cell lymphoma is associated with favorable clinical outcome. *Blood Adv* 2022; Blood Adv.

Hershko DD. Oncogenic properties and prognostic implications of the ubiquitin ligase Skp2 in cancer. *Cancer* [Internet] 2008;**112**:1415–1424. Cancer.

Howard JH, Frolov A, Tzeng CWD, Stewart A, Midzak A, Majmundar A, Godwin AK, Heslin MJ, Bellacosa A, Arnoletti JP. Epigenetic downregulation of the DNA repair gene MED1/MBD4 in colorectal and ovarian cancer. *Cancer Biol Ther* [Internet] 2009;**8**:1. NIH Public Access.

Hu H-L, Wang C-F, Wei X-H, Lv J-X, Cao X-H, Shi Y-Y, Han L-F, Zhang Y-N. Correlation between procollagen-lysine, 2-oxoglutarate 5-dioxygenase 2 and breast cancer. *Int J Clin Exp Pathol* 2019;**12**:1015–1021.

Janaki Ramaiah M, Vaishnave S. BMI1 and PTEN are key determinants of breast cancer therapy: A plausible therapeutic target in breast cancer. *Gene* [Internet] 2018;**678**:302–311. Gene.

Jardim DL, Millis SZ, Ross JS, Woo MS-A, Ali SM, Kurzrock R. Cyclin Pathway Genomic Alterations Across 190,247 Solid Tumors: Leveraging Large-Scale Data to Inform Therapeutic Directions. *Oncologist* [Internet] 2021;**26**:e78–e89. Oncologist.

Jayadev S, Bird TD. Hereditary ataxias: overview. *Genet Med* 2013;**15**:673–683. Genet Med.

Jones RH, Vasey PA. New directions in testicular cancer; molecular determinants of oncogenesis and treatment success. *Eur J Cancer* [Internet] 2003;**39**:147–156. Eur J Cancer.

Kaikkonen E, Takala A, Pursiheimo JP, Wahlström G, Schleutker J. The interactome of the prostate-specific protein Anoctamin 7. *Cancer Biomarkers* 2020;**28**:91. IOS Press.

Katoh M, Igarashi M, Fukuda H, Nakagama H, Katoh M. Cancer genetics and genomics of human FOX family genes. *Cancer Lett* [Internet] 2013;**328**:198–206. Cancer Lett.

Lai Y, Xu P, Wang J, Xu K, Wang L, Meng Y. Tumour suppressive long non-coding RNA AFDN-DT inhibits gastric cancer invasion via transcriptional regulation. *J Cell Mol Med* [Internet] 2020;**24**:3157–3166. J Cell Mol Med.

Leiner J, Loarer F Le. The current landscape of rhabdomyosarcomas: an update. *Virchows Arch* 2020;**476**:97–108. Virchows Arch.

Leonard M, Zhang X. Estrogen receptor coactivator Mediator Subunit 1 (MED1) as a tissue-specific therapeutic target in breast cancer. *J Zhejiang Univ Sci B* [Internet] 2019;**20**:381–390. J Zhejiang Univ Sci B.

Liang S, Guo H, Ma K, Li X, Wu D, Wang Y, Wang W, Zhang S, Cui Y, Liu Y, *et al.* A PLCB1-PI3K-AKT Signaling Axis Activates EMT to Promote Cholangiocarcinoma Progression. *Cancer Res* 2021;**81**:5889–5903. American Association for Cancer Research Inc.

Lin D, Fu Z, Yang G, Gao D, Wang T, Liu Z, Li G, Wang Y. Exportin-5 SUMOylation promotes hepatocellular carcinoma progression. *Exp Cell Res* [Internet] 2020;**395**:. Exp Cell Res.

Liu X, Wei W, Li X, Shen P, Ju D, Wang Z, Zhang R, Yang F, Chen C, Cao K, *et al.* BMI1 and MEL18 Promote Colitis-Associated Cancer in Mice via REG3B and STAT3. *Gastroenterology* [Internet] 2017;**153**:1607–1620. Gastroenterology.

Liu Y, Boukhelifa M, Tribble E, Morin-Kensicki E, Uetrecht A, Bear JE, Bankaitis VA. The Sac1 phosphoinositide phosphatase regulates Golgi membrane morphology and mitotic spindle organization in mammals. *Mol Biol Cell* 2008;**19**:3080–3096. Mol Biol Cell.

Lu ML, Zhang Y, Li J, Fu Y, Li WH, Zhao GF, Li XH, Wei L, Liu GB, Huang H. MicroRNA-124 inhibits colorectal cancer cell proliferation and suppresses tumor growth by interacting with PLCB1 and regulating Wnt/β-catenin signaling pathway. *Eur Rev Med Pharmacol Sci* 2019;**23**:121–136. Verduci Editore s.r.l.

Lucci-Cordisco E, Neri G. Silent beginning: early silencing of the MED1/MBD4 gene in colorectal tumorigenesis. *Cancer Biol Ther* [Internet] 2009;**8**:192–193. Cancer Biol Ther.

MacLeod G, Varmuza S. Tandem affinity purification in transgenic mouse embryonic stem cells identifies DDOST as a novel PPP1CC2 interacting protein. *Biochemistry* 2012;**51**:9678–9688. American Chemical Society.

Messai Y, Noman MZ, Hasmim M, Janji B, Tittarelli A, Boutet M, Baud É, Viry E, Billot K, Nanbakhsh A, *et al.* ITPR1 protects renal cancer cells against natural killer cells by inducing autophagy. *Cancer Res* 2014;**74**:6820–6832. Cancer Res.

Mirzaa GM, Conway RL, Gripp KW, Lerman-Sagie T, Siegel DH, deVries LS, Lev D, Kramer N, Hopkins E, Graham JM, *et al.* Megalencephaly-capillary malformation (MCAP) and megalencephaly-polydactyly-polymicrogyria-hydrocephalus (MPPH) syndromes: two closely related disorders of brain overgrowth and abnormal brain and body morphogenesis. *Am J Med Genet A* [Internet] 2012;**158A**:269–291. Am J Med Genet A.

Moon AL, Haan N, Wilkinson LS, Thomas KL, Hall J. CACNA1C: Association With Psychiatric Disorders, Behavior, and Neurogenesis. *Schizophr Bull* 2018;**44**:958–965. Schizophr Bull.

Mori T, Nishimura N, Hasegawa D, Kawasaki K, Kosaka Y, Uchide K, Yanai T, Hayakawa A, Takeshima Y, Nishio H, *et al.* Persistent detection of a novel MLL-SACM1L rearrangement in the absence of leukemia. *Leuk Res* 2010;**34**:1398–1401. Leuk Res.

Nagase M, Fujita T. Role of Rac1-mineralocorticoid-receptor signalling in renal and cardiac disease. *Nat Rev Nephrol* 2013;**9**:86–98. Nat Rev Nephrol.

Panagopoulos I, Andersen K, Eilert-Olsen M, Zeller B, Munthe-Kaas MC, Buechner J, Osnes LTN, Micci F, Heim S. Therapy-induced Deletion in 11q23 Leading to Fusion of KMT2A With ARHGEF12 and Development of B Lineage Acute Lymphoplastic Leukemia in a Child Treated for Acute Myeloid Leukemia Caused by t(9;11)(p21;q23)/ KMT2A-MLLT3. *Cancer Genomics Proteomics* 2021;**18**:67–81. Cancer Genomics Proteomics.

Pinyol R, Torrecilla S, Wang H, Montironi C, Piqué-Gili M, Torres-Martin M, Wei-Qiang L, Willoughby CE, Ramadori P, Andreu-Oller C, *et al.* Molecular characterisation of hepatocellular carcinoma in patients with non-alcoholic steatohepatitis. *J Hepatol* 2021;**75**:865–878. J Hepatol.

Pulvino M, Liang Y, Oleksyn D, DeRan M, Pelt E Van, Shapiro J, Sanz I, Chen L, Zhao J. Inhibition of proliferation and survival of diffuse large B-cell lymphoma cells by a small-molecule inhibitor of the ubiquitin-conjugating enzyme Ubc13-Uev1A. *Blood* 2012;**120**:1668–1677. Blood.

Qiao Z, Jiang Y, Wang L, Wang L, Jiang J, Zhang J. Mutations in KIAA1109, CACNA1C, BSN, AKAP13, CELSR2, and HELZ2 Are Associated With the Prognosis in Endometrial Cancer. *Front Genet* 2019;**10**:909. Frontiers Media S.A.

Rangel R, Guzman-Rojas L, Kodama T, Kodama M, Newberg JY, Copeland NG, Jenkins NA. Identification of New Tumor Suppressor Genes in Triple-Negative Breast Cancer. *Cancer Res* 2017;**77**:4089–4101. Cancer Res.

Rasheed SAK, Leong HS, Lakshmanan M, Raju A, Dadlani D, Chong FT, Shannon NB, Rajarethinam R, Skanthakumar T, Tan EY, *et al.* GNA13 expression promotes drug resistance and tumor-initiating phenotypes in squamous cell cancers. *Oncogene* 2018;**37**:1340–1353. Oncogene.

Rasheed SAK, Teo CR, Beillard EJ, Voorhoeve PM, Casey PJ. MicroRNA-182 and microRNA-200a control G-protein subunit α-13 (GNA13) expression and cell invasion synergistically in prostate cancer cells. *J Biol Chem* 2013;**288**:7986–7995. J Biol Chem.

Reijnders MRF, Ansor NM, Kousi M, Yue WW, Tan PL, Clarkson K, Clayton-Smith J, Corning K, Jones JR, Lam WWK, *et al.* RAC1 Missense Mutations in Developmental Disorders with Diverse Phenotypes. *Am J Hum Genet* 2017;**101**:466. Elsevier.

Remacha L, Currás-Freixes M, Torres-Ruiz R, Schiavi F, Torres-Pérez R, Calsina B, Letón R, Comino-Méndez I, Roldán-Romero JM, Montero-Conde C, *et al.* Gain-of-function mutations in DNMT3A in patients with paraganglioma. *Genet Med* 2018;**20**:1644–1651. Genet Med.

Remmerie M, Janssens V. PP2A: A Promising Biomarker and Therapeutic Target in Endometrial Cancer. *Front Oncol* 2019;**9**:462. Frontiers Media SA.

Sablina AA, Chen W, Arroyo JD, Corral L, Hector M, Bulmer SE, DeCaprio JA, Hahn WC. The tumor suppressor PP2A Abeta regulates the RalA GTPase. *Cell* 2007;**129**:969–982. Cell.

Shi L, Yan Y, He Y, Yan B, Pan Y, Orme JJ, Zhang J, Xu W, Pang J, Huang H. Mutated SPOP E3 ligase promotes 17βHSD4 protein degradation to drive androgenesis and prostate cancer progression. *Cancer Res* [Internet] 2021;**81**:3593–3606. American Association for Cancer Research Inc.

Sicinski P, Donaher JL, Geng Y, Parker SB, Gardner H, Park MY, Robker RL, Richards JAS, McGinnis LK, Biggers JD, *et al.* Cyclin D2 is an FSH-responsive gene involved in gonadal cell proliferation and oncogenesis. *Nature* [Internet] 1996;**384**:470–474. Nature.

Sogawa K, Yamada T, Sugita A, Kito K, Tachibana M, Nezu K, Ueda N. Role of protein phosphatase in malignant osteogenic and soft tissue tumors. *Res Commun Mol Pathol Pharmacol* 1996;**93**:33–42.

Song TT, Xu F, Wang W. Inhibiting ubiquitin conjugating enzyme E2 N by microRNA-590-3p reduced cell growth of cervical carcinoma. *Kaohsiung J Med Sci* 2020;**36**:501–507. Kaohsiung J Med Sci.

Splawski I, Timothy KW, Sharpe LM, Decher N, Kumar P, Bloise R, Napolitano C, Schwartz PJ, Joseph RM, Condouris K, *et al.* Ca(V)1.2 calcium channel dysfunction causes a multisystem disorder including arrhythmia and autism. *Cell* 2004;**119**:19–31. Cell.

Springelkamp H, Iglesias AI, Cuellar-Partida G, Amin N, Burdon KP, Leeuwen EM van, Gharahkhani P, Mishra A, Lee SJ van der, Hewitt AW, *et al.* ARHGEF12 influences the risk of glaucoma by increasing intraocular pressure. *Hum Mol Genet* 2015;**24**:2689–2699. Hum Mol Genet.

Sun TT, Wang Y, Cheng H, Zhang XH, Xiang JJ, Zhang JT, Yu SBS, Martin TA, Ye L, Tsang LL, *et al.* Disrupted interaction between CFTR and AF-6/afadin aggravates malignant phenotypes of colon cancer. *Biochim Biophys Acta* [Internet] 2014;**1843**:618–628. Biochim Biophys Acta.

Takai Y, Nakanishi H. Nectin and afadin: novel organizers of intercellular junctions. *J Cell Sci* [Internet] 2003;**116**:17–27. J Cell Sci.

Taylor BS, Schultz N, Hieronymus H, Gopalan A, Xiao Y, Carver BS, Arora VK, Kaushik P, Cerami E, Reva B, *et al.* Integrative genomic profiling of human prostate cancer. *Cancer Cell* 2010;**18**:11–22. Cancer Cell.

Tovy A, Rosas C, Gaikwad AS, Medrano G, Zhang L, Reyes JM, Huang Y-H, Arakawa T, Kurtz K, Conneely SE, *et al.* Perturbed hematopoiesis in individuals with germline DNMT3A overgrowth Tatton-Brown-Rahman syndrome. *Haematologica* 2022;**107**:0–0. Haematologica.

Wan J, Qin J, Cao Q, Hu P, Zhong C, Tu C. Hypoxia-induced PLOD2 regulates invasion and epithelial-mesenchymal transition in endometrial carcinoma cells. *Genes Genomics* [Internet] 2020;**42**:317–324. Genes Genomics.

Wang CY, Lai MD, Phan NN, Sun Z, Lin YC. Meta-Analysis of Public Microarray Datasets Reveals Voltage-Gated Calcium Gene Signatures in Clinical Cancer Patients. *PLoS One* 2015;**10**:e0125766. Public Library of Science.

Wang J, Qian J, Hoeksema MD, Zou Y, Espinosa A V., Rahman SMJ, Zhang B, Massion PP. Integrative genomics analysis identifies candidate drivers at 3q26-29 amplicon in squamous cell carcinoma of the lung. *Clin Cancer Res* 2013;**19**:5580–5590. Clin Cancer Res.

Wang R, Xue X, Wang Y, Zhao H, Zhang Y, Wang H, Miao D. BMI1 Deficiency Results in Female Infertility by Activating p16/p19 Signaling and Increasing Oxidative Stress. *Int J Biol Sci* [Internet] 2019;**15**:870–881. Int J Biol Sci.

Wang SS, Esplin ED, Li JL, Huang L, Gazdar A, Minna J, Evans GA. Alterations of the PPP2R1B gene in human lung and colon cancer. *Science* 1998;**282**:284–287. Science.

Wei X, Lv H, Yang S, Yang X. CircRNA PLOD2 enhances ovarian cancer propagation by controlling miR-378. *Saudi J Biol Sci* [Internet] 2021;**28**:6260–6265. Saudi J Biol Sci.

Whaley NR, Fujioka S, Wszolek ZK. Autosomal dominant cerebellar ataxia type I: a review of the phenotypic and genotypic characteristics. *Orphanet J Rare Dis* 2011;**6**:. Orphanet J Rare Dis.

Wu L, Zhu J, Liu D, Sun Y, Wu C. An integrative multiomics analysis identifies putative causal genes for COVID-19 severity. *Genet Med* 2021a;**23**:2076–2086. Elsevier.

Wu T, Gu X, Cui H. Emerging Roles of SKP2 in Cancer Drug Resistance. *Cells* [Internet] 2021b;**10**:. Cells.

Wu X, Zhang W, Font-Burgada J, Palmer T, Hamil AS, Biswas SK, Poidinger M, Borcherding N, Xie Q, Ellies LG, *et al.* Ubiquitin-conjugating enzyme Ubc13 controls breast cancer metastasis through a TAK1-p38 MAP kinase cascade. *Proc Natl Acad Sci U S A* 2014;**111**:13870–13875. Proc Natl Acad Sci U S A.

Xu Y, Rong J, Duan S, Chen C, Li Y, Peng B, Yi B, Zheng Z, Gao Y, Wang K, *et al.* High expression of GNA13 is associated with poor prognosis in hepatocellular carcinoma. *Sci Reports 2016 61* 2016;**6**:1–10. Nature Publishing Group.

Yagi H, Asanoma K, Ohgami T, Ichinoe A, Sonoda K, Kato K. GEP oncogene promotes cell proliferation through YAP activation in ovarian cancer. *Oncogene 2016 3534* 2016;**35**:4471–4480. Nature Publishing Group.

Yamada T, Sogawa K, Masaki T, Funamoto Y, Kohno K, Oka S, Norimatsu H, Matsumoto K. Enhanced expression of catalytic subunit isoform PP1γ1 of protein phosphatase type 1 in malignant fibrous histiocytoma. *Res Commun Mol Pathol Pharmacol* 1994;**86**:125–128.

Yamamoto T, Mori T, Sawada M, Matsushima H, Ito F, Akiyama M, Kitawaki J. Loss of AF-6/afadin induces cell invasion, suppresses the formation of glandular structures and might be a predictive marker of resistance to chemotherapy in endometrial cancer. *BMC Cancer* [Internet] 2015;**15**:. BMC Cancer.

Zhang JX, Yun M, Xu Y, Chen JW, Weng HW, Zheng ZS, Chen C, Xie D, Ye S. GNA13 as a prognostic factor and mediator of gastric cancer progression. *Oncotarget* 2016a;**7**:4414–4427. Oncotarget.

Zhang W, Cao L, Sun Z, Xu J, Tang L, Chen W, Luo J, Yang F, Wang Y, Guan X. Skp2 is over-expressed in breast cancer and promotes breast cancer cell proliferation. *Cell Cycle* [Internet] 2016b;**15**:1344–1351. Cell Cycle.

Zhang Z, Tan X, Luo J, Cui B, Lei S, Si Z, Shen L, Yao H. GNA13 promotes tumor growth and angiogenesis by upregulating CXC chemokines via the NF-κB signaling pathway in colorectal cancer cells. *Cancer Med* 2018;**7**:5611–5620. Cancer Med.

Zhao Q, Qian Q, Cao D, Yang J, Gui T, Shen K. Role of BMI1 in epithelial ovarian cancer: investigated via the CRISPR/Cas9 system and RNA sequencing. *J Ovarian Res* [Internet] 2018;**11**:. J Ovarian Res.
